# Epidemiological characteristics and trends of notifiable infectious diseases in China from 1986 to 2016

**DOI:** 10.7189/jogh.10.020803

**Published:** 2020-12

**Authors:** Yi Jiang, Xiangfeng Dou, Chenqi Yan, Li Wan, Haican Liu, Machao Li, Ruibai Wang, Guilian Li, Lili Zhao, Zhiguang Liu, Xiuqin Zhao, Kanglin Wan

**Affiliations:** 1 State Key Laboratory for Infectious Disease Prevention and Control, National Institute for Communicable Disease Control and Prevention, Collaborative Innovation Center for Diagnosis and Treatment of Infectious Diseases, Chinese Center for Disease Control and Prevention, Beijing, China; 2 Beijing Center for Diseases Prevention and Control, Beijing, China; 3 Foshan Women and Children Hospital, Guangdong Province, China; 4 Department of Physiology, Xiangya School of Medicine, Central South University, Changsha, China

## Abstract

**Background:**

Since the 1980s, China has undergone significant social change and the incidence of infectious diseases has also changed considerably. Here, we report the epidemiological features and changes in notifiable infectious diseases in China from 1986 to 2016 to explore the factors contributing to the successful control of infectious diseases and the challenges faced in the prevention and control of infectious diseases.

**Methods:**

The data of notifiable infectious diseases in China from 1986 to 2016 were collected from the monthly analysis report of the National Infectious Disease Surveillance System. Joinpoint regression models were used to examine incidence and mortality trends from 1986 to 2016. IBM SPSS Statistics version 22.0, Excel 2010 and R x64 3.5.2 were used for data analysis.

**Results:**

A total of 132 858 005 cases of notifiable infectious diseases were reported over these 31 years, with an average yearly incidence of 342.14/100 000. There were 284 694 deaths with an average yearly mortality rate of 0.73/100 000. The overall incidence and overall mortality of notifiable infectious diseases both showed a “U” distribution (ie, a decrease, stable, an increase, stable again). The top five diseases in terms of incidence were hand, foot and mouth disease, viral hepatitis, tuberculosis, other infectious causes of diarrhea and dysentery, accounting for 78.0% of all reported cases. The top five causes of death were HIV/AIDS, rabies, tuberculosis, viral hepatitis and epidemic encephalitis B, which accounted for 76.07% of all mortalities. The diseases with the top five fatality rates were rabies, H5N1, H7N9, HIV/AIDS and plague, with rates of 91.06%, 66.07%, 38.51%, 25.19% and 10.31%, respectively.

**Conclusions:**

This analysis will benefit the future monitoring of infectious diseases and public health measures in China.

The occurrence of infectious diseases is not only related to the source of infection, the route of transmission and the susceptible population, but is also affected by natural and social factors. Since the 1980s, China has undergone tremendous social changes [1,2], and the incidence of infectious diseases has changed considerably as a result of improvements in sanitary conditions and the nutritional status of the population, advances in diagnostic and therapeutic techniques, and the increased application of vaccines and drugs. By the year 2000, the incidence of infectious diseases in China had dropped significantly [3]. Most infectious diseases had been effectively controlled, and some had even been eliminated. However, as a result of the prevention and control of infectious diseases abating, traditional infectious diseases such as tuberculosis re-emerged and became epidemic, and new infectious diseases such as severe acute respiratory syndrome (SARS) [4], avian influenza A H5N1[5], poliomyelitis [6], pandemic H1N1 influenza [7] and avian influenza A H7N9 [8] also emerged and became epidemic. This has highlighted the fact that infectious diseases are still a major disease burden in China [9].

In China, a national standardized reporting system for selected infectious diseases was established in the 1950s [3]. From the 1980s onwards, the data collected, using this system, have become more consistent and reliable. Only two major changes have been made to this system over time. First, the number of notiﬁable diseases in the system has increased during this time. Second, the method of reporting has changed, switching from paper-based reporting in 1950 to an electronic version of the report in 1985, and then an internet-based case reporting system was introduced in 2003. This study selects the annual data from the 1986 to 2016 infectious disease surveillance system report to describe the epidemiological characteristics and trends of infectious diseases in China over the past 31 years. The factors contributing to the successful control of infectious diseases are explored, along with the challenges faced in the prevention and control of infectious diseases.

## METHODS

### Data sources

The data come from the monthly analysis report of the National Infectious Disease Surveillance System from 1986 to 2016, which was reported by the Division of Infectious Disease, Chinese Center for Disease Control and Prevention. The data includes the numbers of cases and deaths per month, and those of the same month in the previous year were also included. The annual population data for the years 1986–2016 were collected from the data published on the website of the Chinese National Bureau of Statistics.

### Procedures

The National Infectious Disease Surveillance System have started from 1955 in China. In 1955, there were 18 notifiable infectious diseases and this number increased to 25 in 1978 [10]. These diseases were divided into two categories: A and B. In 1989, the first “Law of the People's Republic of China on the Prevention and Treatment of Infectious Diseases” stipulated 35 notifiable infectious diseases, which were classified into classes A, B and C. The numbers of notifiable infectious diseases increased over time. Currently, there are totaling 39 notifiable infectious diseases in China. This equated to a total of 45 notifiable infectious diseases over time in China (**Table 1**).

**Table 1:** Changes to the list of notifiable diseases in China, 1955-2016

Year	No. of diseases	Newly added	Cancel-reporting
1955	18	Plague, cholera, smallpox, epidemic encephalitis B, diphtheria, scrubtyphus, relapsing fever, dysentery, typhoid fever, scarlet fever, epidemic cerebrospinal meningitis (ECM), measles, poliomyelitis, pertussis, anthrax, undulant fever, forest encephalitis and rabies	-
1978	25	Influenza, viral hepatitis, Tsutsugamushi disease, epidemic haemorrhagic fever (EHF), leptospirosis, brucellosis, malaria	-
1989	35	HIV/AIDS, gonorrhea, syphilis, kala-azar, dengue fever, tuberculosis, schistosomiasis, filariasis, echinococcosis, lepra, epidemic parotitis, rubella, neonatal tetanus, acute hemorrhagic conjunctivitis (AHC), other infectious diarrhea*	Smallpox, relapsing fever, forest encephalitis, Tsutsugamushi disease, undulant fever
2004	37	SARS and highly pathogenic avian influenza A (H5N1)	-
2008	38	Hand, foot and mouth disease	-
2009	39	Influenza A H1N1	-
2013	39	H7N9 avian influenza	Influenza A H1N1

*Other infectious diarrhea refers to diarrhea other than that caused by cholera, dysentery or typhoid fever.

The monitoring of three infectious diseases, that is, smallpox, relapsing fever and undulant fever, was stopped in 1989, after no incidences were reported between 1986 and 1989. Therefore, this study did not include the analysis of these diseases but did analyze the remaining 42 diseases. Influenza A H1N1 was only reported in 2009-2013, As H1N1 was included in the monitoring of influenza viruses in 2014, this study combined the H1N1 influenza data with those of other influenza viruses for statistical analysis.

### Statistical analysis

Yearly mortality (per 100 000) was the number of deaths per year divided by the mid-year population size. The fatality rate (per 100) was the number of reported deaths divided by the number of reported cases over the same period.

We used joinpoint regression models [11] to examine incidence and mortality trends from 1986 to 2016 and to identify changes in trends at every stage. The software Joinpoint Regression Program Version 4.7.0.0 (Statistical Research and Applications Branch, National Cancer Institute) was employed to build the joinpoint regression models, calculate annual percentage changes (APC) and average annual percentage changes (AAPC) to express trends. A z test or *t* test was used to assess whether an APC or AAPC was significantly different from zero. The terms increase and decrease were used when the slope was significant (*P* < 0.05). A non-significant annual percentage change (*P* ≥ 0.05) was described as stable, indicating that the incidence was maintained at a perennially stable level or that the incidence was perennially unreported or only reported sporadically.

We used IBM SPSS Statistics version 22.0 (IBM, Armonk, NY, USA) and Excel 2010 (Microsoft, Seattle WA, USA) to extract, sort and clean data. IBM SPSS Statistics v22.0 and R x64 3.5.2 (Foundation for Statistical Computing, Vienna, Austria) were used for further data analysis.

## RESULTS

### Overall incidence and mortality rate

Between 1986 and 2016, a total of 42 infectious diseases were included in our study. As shown in **Table 2**, a total of 132 858 005 cases of infectious diseases were reported in these 31 years, with an average yearly incidence of 342.14/100 000. Among the reported cases of infectious diseases, there were 284 694 deaths from notifiable infectious diseases, with an average yearly mortality rate of 0.73/100 000 (**Table 2**).

**Table 2:** Incidence, mortality and fatality rates of notifiable diseases in 1986-2016

Year	No. of notifiable diseases	Mid-year population (10000)	Cases of notifiable infectious diseases	Incidence (/100 000)	Death number	Mortality (/100 000)	Fatality (%)
1986	25	106 679	7 210 479	675.90	12530	1.17	0.17
1987	25	108 404	5 350 224	493.55	10836	1.00	0.20
1988	25	110 163	4 534 327	411.60	12739	1.16	0.28
1989	35	111 865	3 224 377	288.24	10692	0.96	0.33
1990	35	113 519	2 912 467	256.56	10446	0.92	0.36
1991	35	115 078	2 965 911	257.73	7146	0.62	0.24
1992	35	116 497	2 496 505	214.30	4995	0.43	0.20
1993	35	117 844	2 025 825	171.91	4229	0.36	0.21
1994	35	119 184	2 124 620	178.26	4353	0.37	0.20
1995	35	120 486	1 967 202	163.27	3102	0.26	0.16
1996	35	121 755	1 847 112	151.71	2965	0.24	0.16
1997	35	123 008	2 243 544	182.39	3867	0.31	0.17
1998	35	124 194	2 403 887	193.56	3839	0.31	0.16
1999	35	125 274	2 433 031	194.22	3351	0.27	0.14
2000	35	126 265	2 302 929	182.39	3241	0.26	0.14
2001	35	127 185	2 337 774	183.81	3599	0.28	0.15
2002	35	128 040	2 312 067	180.57	4484	0.35	0.19
2003	35	128 840	2 508 234	194.68	5685	0.42	0.21
2004	37	129 608	3 482 492	268.70	5526	0.43	0.16
2005	37	130 372	3 966 853	304.27	8088	0.62	0.20
2006	37	131 102	4 110 606	313.54	8457	0.65	0.21
2007	37	131 789	4 839 527	367.22	7868	0.60	0.16
2008	38	132 466	5 874 475	443.47	11086	0.84	0.19
2009	39	133 126	6 562 737	492.97	12888	0.97	0.20
2010	39	133 771	7 066 563	528.26	15855	1.19	0.22
2011	39	134 413	6 999 486	520.74	16558	1.23	0.24
2012	39	135 070	7 658 424	567.00	17618	1.30	0.23
2013	39	135 738	7 068 165	520.72	16399	1.21	0.23
2014	39	136 427	7 688 792	563.58	16287	1.19	0.21
2015	39	137 122	6 847 852	499.40	18057	1.32	0.26
2016	39	137 867	7 491 518	543.39	17908	1.30	0.24

### Trends and changes in yearly incidence

**Figure 1:**
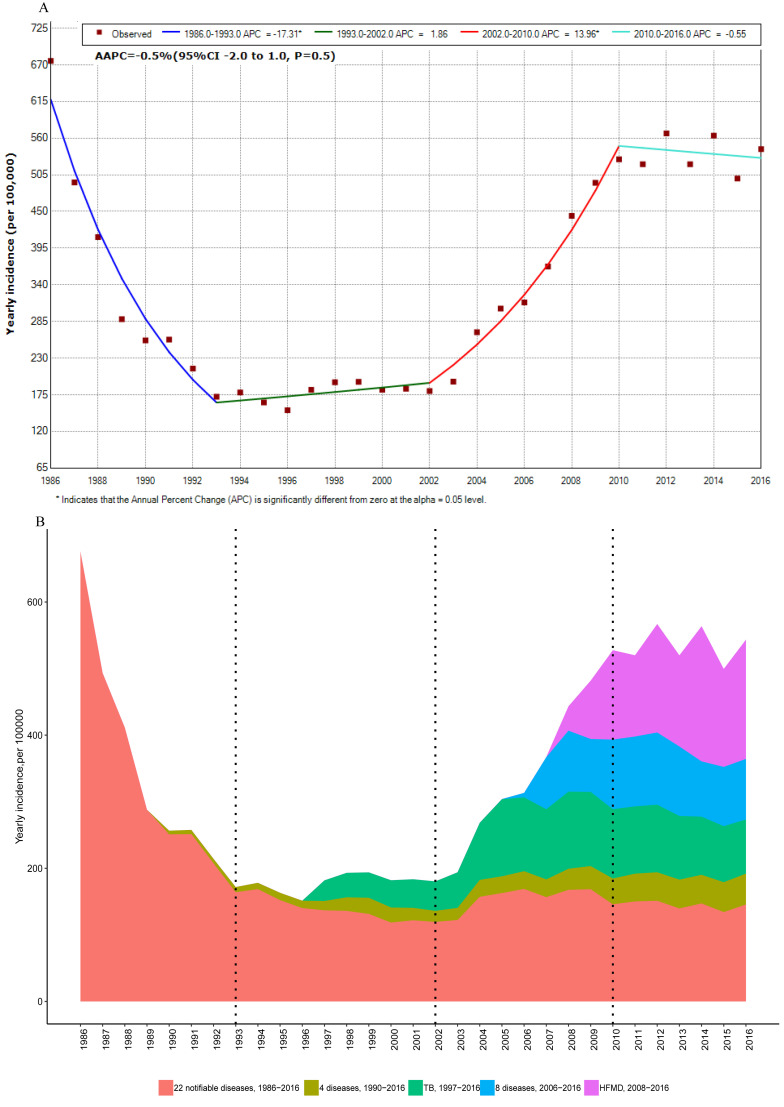
Trends in all incidences of notifiable diseases by year for 1986-2016 in China **Panel A.** Joinpoint regression showing trends in overall incidence of 42 notifiable diseases. Three joinpoints were detected: 1993, 2002 and 2010. Panel B. Proportions of 36 notifiable diseases from the overall incidence from 1986 to 2016. 22 notifiable diseases, 1986-2016: Plague, cholera, epidemic encephalitis B, diphtheria, scrub typhus, dysentery, typhoid fever, scarlet fever, epidemic cerebrospinal meningitis (ECM), measles, poliomyelitis, pertussis, anthrax, rabies, influenza, viral hepatitis, ebola hemorrhagic fever (EHF), leptospirosis, brucellosis, malaria, kala-azar, filariasis. 4 notifiable diseases, 1990-2016: AIDS, dengue fever, gonorrhea, syphilis. 8 notifiable diseases, 2006-2016: Echinococcosis, rubella, acute hemorrhagic conjunctivitis (AHC), epidemic parotitis, lepra, other infectious diarrhea, H5N1, schistosomiasis. The proportions of the other six diseases were small so are not presented in panel B.

During the three decades, the yearly incidence of all notifiable infectious diseases presented a “U”-shaped distribution (F**igure 1**, Panel A). The AAPC of all notifiable infectious diseases was 0.5% (95% confidence interval (CI) = -2.0 to 1.0, *P* = 0.7) with the highest yearly incidence of 675.90/100 000 in 1986. Joinpoint regression indicated that the trends in APC of yearly incidence could be divided into four stages. From 1986 to 1993, the APC decreased year by year, and was -17.3% (95% CI = -19.8 to -14.2, *P* < 0.05). From 1993 to 2002, the APC was stable at a low level. From 2002 to 2010, the APC increased significantly, and was 14.0% (95% CI = 10.6 to 17.5, *P* < 0.05). After 2010, the APC was stable at a higher level. The detailed results are presented in **Figure 1**, Panel A.

The proportion of yearly incidence can be seen in **Figure 1**, Panel B. The yearly incidence of 22 consistently reported infectious diseases in 1986–2016 continued to decline from 1986 to 1993 (APC = -12.4%, 95% CI = -14.0 to -10.8), and were stably maintained at a lower level from 1993 to 2016 (AAPC = -1.5%, 95% CI = -3.9 to 1.0). Among the 22 diseases, the proportions of viral hepatitis, dysentery and influenza were the highest, at 48.56%, 29.62% and 8.61%, respectively. From 1990, the yearly incidence of syphilis and HIV/AIDS among the four newly reported diseases (AIDS, dengue fever, gonorrhea and syphilis) increased significantly. The yearly incidence of tuberculosis continued to increase after 1997. Among the eight newly reported diseases in 2006, the proportions of other infectious diarrhea, mumps, acute hemorrhagic conjunctivitis and rubella were 66.81%, 23.84%, 4.49% and 3.71%, respectively. Except for other infectious diarrhea, the yearly incidence of the other three diseases was stable or decreased. The yearly incidence of hand, foot and mouth disease continued to increase after 2008. These newly reported diseases have caused an increase in the yearly incidence of all notifiable infectious diseases since 2002.

**Figure 2:**
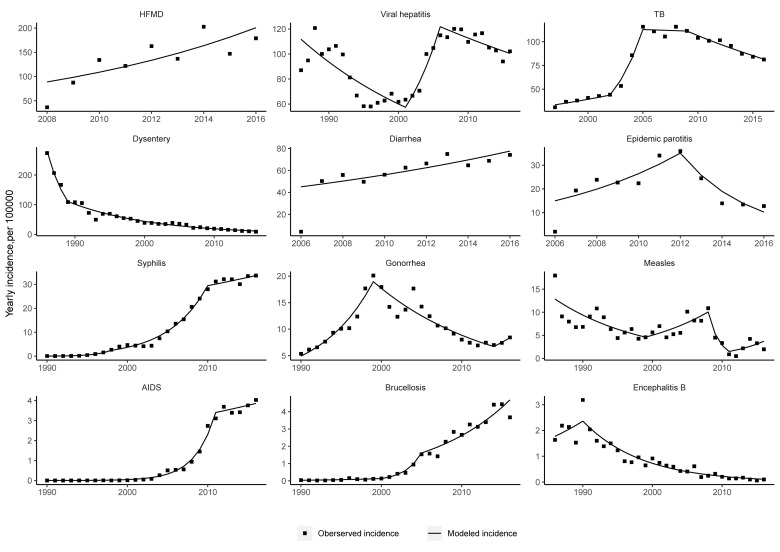
Trends in the incidences of 12 notifiable diseases by year from 1986-2016 in China.Yearly APC values for incidence and overall trends are shown for 1986 to 2016. Squares denote the observed values and lines denote the slope of the APC.

**Figure 3:**
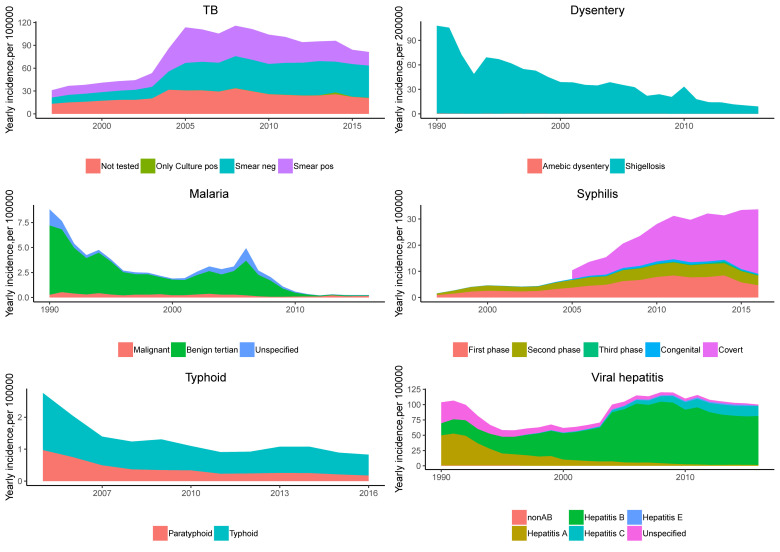
Different types of cases among nine notifiable diseases.

The incidence of viral hepatitis was relatively stable. The incidence of viral hepatitis declined up to 2001, increased rapidly between 2001 and 2006 (APC = 16.2%, 95% CI = 4.1 to 29.8), then stabilized at a high level (APC = -1.9%, 95% CI = -4.2 to 0.4) (**Figure 2**). The decline before 2001 was due to a decrease in the incidence of hepatitis A, and the subsequent increase was due to a rise in hepatitis B and hepatitis C (**Figure 3**).

The incidence of HIV/AIDS showed an increasing trend across the whole study period. Before 2011, the incidence of HIV/AIDS increased rapidly, and thereafter the incidence stabilized. Syphilis showed the same trend as HIV/AIDS, the incidence of syphilis increased after 2005 mainly because of a rise in covert-type syphilis (**Figure 3**).

The yearly incidence of dysentery decreased significantly. Dysentery, including 99.38% bacterial dysentery and 0.62% amoebic dysentery, both presented a downward trend (see **Figure 3**).

### Trends and changes in yearly mortality

**Figure 4:**
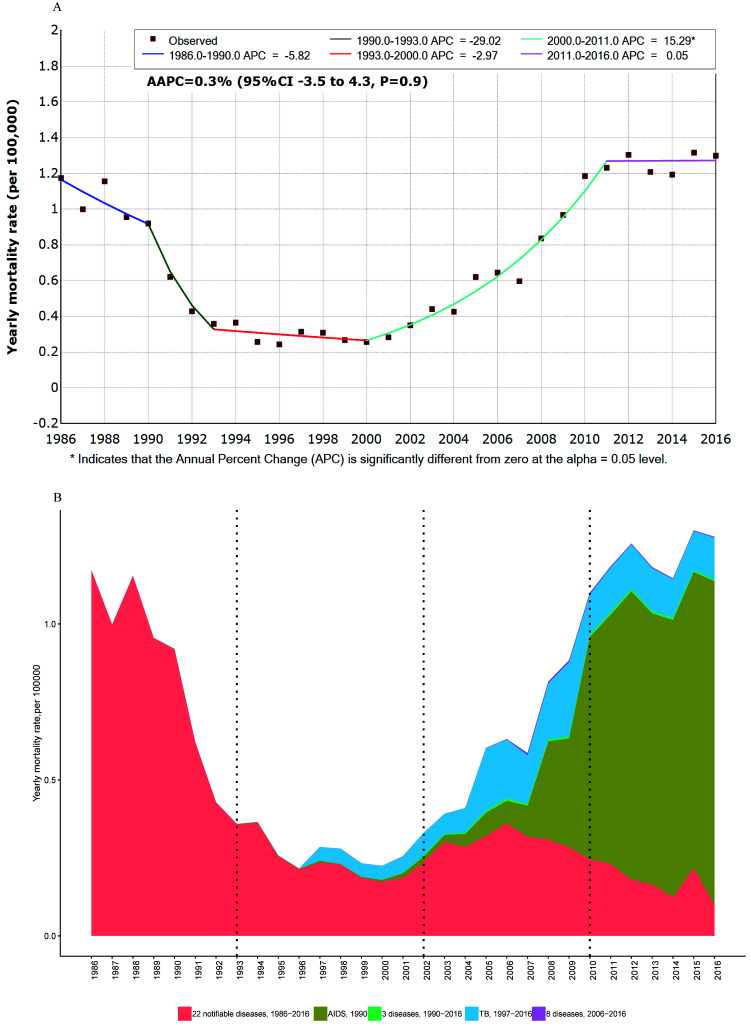
Trends in the mortality rates of notifiable diseases by year from 1986-2016 in China. **Panel A.** Joinpoint regression showing trends in the overall mortality rate of 42 notifiable diseases. Four joinpoints were detected: 1990, 1993, 2000 and 2011. **Panel B.** Proportions of 35 notifiable diseases from the overall mortality rate from 1986 to 2016. 22 notifiable diseases, 1986-2016: Plague, cholera, epidemic encephalitis B, diphtheria, scrubtyphus, dysentery, typhoid fever, scarlet fever, ECM, measles, poliomyelitis, pertussis, anthrax, rabies, influenza, viral hepatitis, EHF, leptospirosis, brucellosis, malaria, kala-azar, filariasis. 3 notifiable diseases, 1990-2016: Dengue fever, gonorrhea, syphilis. 8 notifiable diseases, 2006-2016: echinococcosis, rubella, AHC, Epidemic parotitis, lepra, other infectious diarrhea, H5N1, schistosomiasis. The proportions of the other seven diseases were small so are not presented in panel B.

The yearly mortality rate between 1986 and 2016 also showed a “U” distribution (**Figure 4**, Panel A), peaking at 1.32/100 000 in 2015. During the study period, the yearly mortality rate of all notifiable diseases was stable (AAPC = 0.3%, 95% CI = -3.5 to 4.3, *P* = 0.9). The joinpoint regression results showed that the yearly mortality trend could be divided into five stages, as shown in **Figure 4**, Panel A. After two declines in 1986–1990 and 1990–1993, the yearly incidence stabilized at a low level in 1993-2000, then increased significantly after 2000-2011, then stabilized again at a high level in 2011-2016.

The yearly mortality rate from 22 consistently reported infectious diseases from 1986-2016, declined from 1986 to 1993 (APC = -14.0%, 95% CI = -19.4 to -8.3, *P* < 0.05), then stabilized at a low level from 1993-2016 (APC = -7.1%, 95% CI = -1.9 to -3.4). Among the 22 diseases, the largest proportion of deaths resulted from rabies and viral hepatitis, which accounted for 30.40% and 19.86%, respectively.

Among the four newly reported diseases from 1990, the proportion of HIV/AIDS was 98.69%, and yearly mortality rate of HIV/AIDS continued to rise. After 1997, the yearly mortality rate from tuberculosis continued to increase. After 2011, HIV/AIDS and tuberculosis were the main causes of death from all notifiable infectious diseases (**Figure 4**, Panel B), accounting for 72.37% and 10.71% of deaths, respectively.

### Ranks of incidence and mortality

The top five diseases in terms of incidences were hand, foot and mouth disease, viral hepatitis, tuberculosis, other infectious diarrhea and dysentery, accounting for 78.0% of all reported cases during the whole study period (**Table 3**). In 1986-1993, 1993-2002, 2002-2010 and 2010-2016, the diseases with the highest incidence were dysentery, viral hepatitis, viral hepatitis, and hand, foot and mouth disease, accounting for 40.12%, 36.25%, 31.73%, and 29.00% of cases, respectively, as shown in **Table 3**.

**Table 3:** Incidence rank of notifiable infectious disease by year during 1986-2016 in China

Ranking of cases	1986-2016		1986-		1993-		2002-		2010-	
**Diseases**	**Proportion (%)**	**Disease**	**Proportion, %**	**Disease**	**Proportion (%)**	**Disease**	**Proportion (%)**	**Disease**	**Proportion (%)**
1	HFMD	12.33	Dysentery	40.12	Viral hepatitis	36.25	Viral hepatitis	31.73	HFMD	29
2	Viral hepatitis	26.98	Viral hepatitis	27.79	Dysentery	29.81	Tuberculosis	28.57	Viral hepatitis	19.93
3	Tuberculosis	15.81	Influenza	17.28	Tuberculosis	12.08	Dysentery	10.19	Tuberculosis	17.49
4	diarrhea	6.38	Malaria	3.38	Gonorrhea	7.51	diarrhea	5.9	diarrhea	12.51
5	Dysentery	16.47	Measles	2.65	Measles	3.31	HFMD	4.58	Syphilis	5.9
6	Influenza	4.78	Typhoid fever	2.58	Typhoid fever	2.85	Gonorrhea	4.14	EP	4.18
7	EP	2.28	EHF	1.33	EHF	2.05	Syphilis	3.81	Malaria	2.6
8	Syphilis	3.43	Leptospirosis	1.06	Malaria	1.64	EP	2.5	Influenza	2.18
9	Gonorrhea	2.79	Scarlet fever	0.9	Syphilis	1.2	Measles	2.33	Gonorrhea	1.41
10	Measles	1.79	Pertussis	0.79	Scarlet fever	0.77	Typhoid fever	0.92	AHC	0.99

HFMD – hand foot and mouth disease, Diarrhea – other infectious diarrhea, EP – epidemic parotitis, EHF – epidemic haemorrhagic fever, AHC – acute hemorrhagic conjunctivitis

Seven infectious diseases showed significant increasing trends in incidence during the study period, including HIV/AIDS, schistosomiasis, syphilis, brucellosis, hand, foot and mouth disease, other infectious diarrheal diseases and tuberculosis (**Table 4**). In particular, HIV/AIDS, schistosomiasis, syphilis and brucellosis showed the fastest increases, with AAPC values of 38.0%, 33.9%, 30.2% and 20.5%, respectively.

**Table 4:** Annual percentage change in incidence for infectious diseases during 1986-2016

Diseases	Reporting period	Trend	Average annual percentage change (95% CI)*
HIV/AIDS	1990-2016	Increase	38.0% (33.0 to 43.2)
Schistosomiasis	2006-2016	Increase	33.9% (10 to 62.9)
Syphilis	1990-2016	Increase	30.2% (24.6 to 36.1)
Brucellosis	1989-2016	Increase	20.5% (10.1 to 31.9)
HFMD	2008-2016	Increase	10.7% (1.6 to 20.7)
Other infectious diarrhea	2006-2016	Increase	5.6% (2.7 to 8.5)
Tuberculosis	1997-2016	Increase	4.8% (2.5 to 7.1)
AHC	2006-2016	Stable	8.1% (-9.3 to 28.9)
Dengue fever	1990-2016	Stable	4.5% (-16.8 to 31.2)
Viral Hepatitis	1986-2016	Stable	1.2% (-0.7 to 3.2)
Scarlet fever	1986-2016	Stable	0.4% (-6.9 to 8.2)
Gonorrhea	1990-2016	Stable	-1.9% (-1.3 to 5.1)
Rabies	1986-2016	Stable	-1.9% (-5.9 to -2.3)
Lepra	2006-2016	Stable	-3.1% (-9.9 to 4.2)
Measles	1986-2016	Stable	-4.2% (-14.6 to 7.6)
Typhoid fever	1986-2016	Decrease	-6.9% (-12.0 to -1.5)
EHF	1986-2016	Decrease	-7.2% (-9.2 to -5.1)
Pertussis	1986-2016	Decrease	-8.0% (-12.8 to -3.0)
Leptospirosis	1986-2016	Decrease	-8.7% (-12.0 to -6.2)
Anthrax	1989-2016	Decrease	-9.31% (-10.4 to -8.2)
Dysentery	1986-2016	Decrease	-10.1% (-12.4 to -7.7)
Epidemic Encephalitis B	1986-2016	Decrease	-10.1% (-13.0 to -7.1)
Malaria	1986-2016	Decrease	-13.7% (-21.5 to -5.1)
Neonatal Tetanus	1996-2016	Decrease	-14.8% (-17.0 to -12.5)
Cholera	1989-2016	Decrease	-14.8% (-26.2 to -1.8)
ECM	1986-2016	Decrease	-16.4% (-20.1 to -12.6)
Rubella	2006-2016	Decrease	-21.7% (-33 to -8.4)

HFMD – hand, foot and mouth disease, AHC – acute hemorrhagic conjunctivitis, EHF – epidemic haemorrhagic fever; ECM – epidemic cerebrospinal meningitis. Diarrhea – other infectious diarrhea, EEB – epidemic encephalitis B, CI – confidence interval

*When the yearly incidence data of the disease contained a zero, AAPC was not calculated.

Twelve infectious diseases showed significant decreasing trends in incidence during the study period. In particular, the incidence of vaccine-preventable diseases such as rubella, epidemic cerebrospinal meningitis and pertussis, neonatal tetanus and epidemic encephalitis B, decreased significantly. The AAPC values of polio and diphtheria were not calculated because of the small number of cases each year (**Table 4**). The incidence of intestinal infectious diseases, including cholera, dysentery and typhoid fever, also decreased significantly, with AAPC values of -14.8%, -10.1% and -6.9%, respectively. Natural epidemic diseases such as malaria, leptospirosis, anthrax and Ebola hemorrhagic fever (EHF) also declined significantly (**Table 4**).

The top five causes of death were HIV/AIDS, rabies, tuberculosis, viral hepatitis and epidemic encephalitis B. Deaths from these five diseases accounted for 76.07% of all deaths caused by the notifiable infectious diseases during 1986-2016. As shown in **Table 5**, the top three causes of death in 1986-1993 were rabies, Epidemic cerebrospinal meningitis (ECM) and epidemic encephalitis B; in 1993-2002, were viral hepatitis, epidemic encephalitis B and EHF; in 2002-2010 were rabies, tuberculosis and HIV/AIDS; and in 2010-2016, were HIV/AIDS, tuberculosis and rabies (See **Table 5**).

**Table 5:** Cause of mortality for notifiable infectious diseases by year during 1986-2016 in China

Ranking of death	1986-2016			1986-		1993-			2002-		2010-	
**Disease**	**Proportion (%)**		**Disease**	**Proportion, %**	**Disease**	**Proportion (%)**		**Disease**	**Proportion (%)**	**Disease**	**Proportion, %**
1	HIV/AIDS	34.22		Rabies	22.54	Viral hepatitis	17.58		Rabies	25.96	HIV/AIDS	70.57
2	Rabies	15.48		ECM	13.89	EEB	10.61		Tuberculosis	24.03	Tuberculosis	10.72
3	Tuberculosis	10.94		EEB	13.79	EHF	10.06		HIV/AIDS	20.29	Rabies	7.23
4	Viral hepatitis	10.11		Dysentery	13.29	Leptospirosis	9.42		Viral hepatitis	13.18	Viral hepatitis	5.58
5	EEB	5.32		Viral hepatitis	11.34	Rabies	9.32		NT	2.59	HFMD	2.57
6	Dysentery	4.53		EHF	10.08	Tuberculosis	8.86		EEB	2.51	EHF	0.55
7	ECM	4.46		Leptospirosis	6.01	Dysentery	7.26		EHF	2.02	Syphilis	0.51
8	EHF	4.31		Measles	3.27	NT	6.85		Dysentery	1.76	EEB	0.36
9	Leptospirosis	2.67		Typhoid fever	2.45	ECM	5.83		ECM	1.51	NT	0.25
10	Measles	1.53		Anthrax	0.49	Measles	4.33		H1N1	0.99	H7N9	0.24

EEB – epidemic encephalitis B, EHF – epidemic haemorrhagic fever, HFMD – hand foot and mouth disease, NT – neonatal tetanus

The yearly mortality rates of the most infectious diseases showed a downward trend (see **Table 6**). However, the yearly mortality rates of HIV/AIDS, syphilis and tuberculosis continued to rise with AAPC values of 45.1%, 22.6% and 8.0%, respectively.

**Table 6:** Annual percentage change in the mortality rate for infectious diseases during 1986-2016

Diseases	Reporting period	Trend	Average Annual percentage change, (95%CI)*
HIV/AIDS	1990-2016	Increase	45.1% (39.3 to 51.1)
Syphilis	1990-2016	Increase	22.6% (18.3 to 27.0)
Tuberculosis	1997-2016	Increase	8.0% (3.4 to 12.8)
Viral hepatitis	1986-2016	Stable	-0.6% (-1.9 to 0.7)
Rabies	1986-2016	Stable	-2.6% (-6.4 to -1.3)
Gonorrhea	1990-2016	Stable	-7.2% (-33.3 to 29.0)
HFMD	2008-2016	Stable	7.2% (-33.4 to 72.4)
Malaria	1986-2016	Decrease	-3.2% (-4.7 to -1.6)
Measles	1986-2016	Decrease	-10.6% (-12.4 to -8.7)
EHF	1986-2016	Decrease	-11.6% (-13.3 to -9.9)
Neonatal tetanus	1996-2016	Decrease	-16.9% (-22.0 to -11.4)
Epidemic encephalitis B	1986-2016	Decrease	-12.5% (-14.3 to -10.7)
Anthrax	1989-2016	Decrease	-14.1% (-16.2 to -11.9)
Typhoid fever	1986-2016	Decrease	-14.4% (-16.0 to -12.9)
Dysentery	1986-2016	Decrease	-17.9% (-22.6 to -12.9)
ECM	1986-2016	Decrease	-19.1% (-24.0 to -13.9)
Leptospirosis	1986-2016	Decrease	-19.9% (-24.2 to -15.4)

HFMD – hand, foot and mouth disease, EHF – epidemic haemorrhagic fever, ECM – epidemic cerebrospinal meningitis. EEB – epidemic encephalitis B, CI – confidence interval

*When the yearly mortality rate of the disease contained a zero, AAPC was not calculated.

### Fatality rate

**Figure 5:**
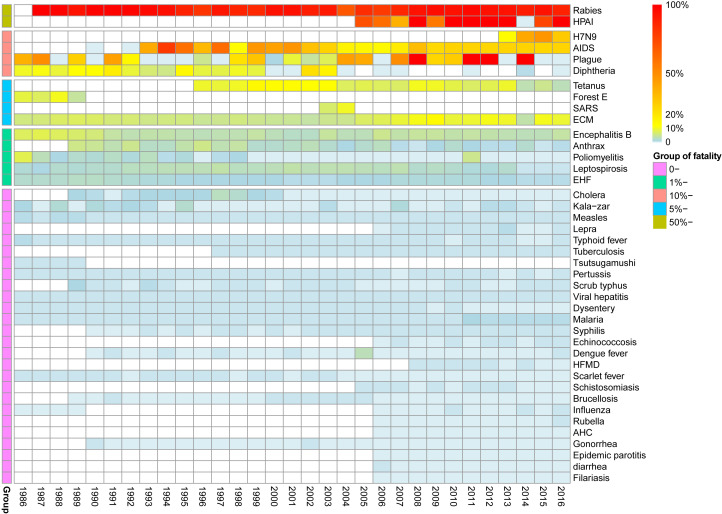
Yearly fatality rates for notifiable infectious diseases by year for 1986-2016 in China. Blanks indicate that no reporting data were available.

The diseases with the top five highest fatality rates were rabies, H5N1, H7N9, HIV/AIDS and the plague, with fatality rates of 91.06%, 66.07%, 38.51%, 25.19% and 10.31%, respectively. Rabies, H5N1, H7N9, HIV/AIDS and the plague maintained high fatality rates during the whole study period (**Figure 5**).

Among the diseases with overall fatality rates between 5% and 10%, the yearly fatality rate of epidemic cerebrospinal meningitis varied from 3.77% to 13.25%, and the yearly fatality rate of neonatal tetanus varied from 2.75 to 13.61%.

The diseases with overall fatality rates of 1%-5% were epidemic encephalitis B, poliomyelitis, leptospirosis, EHF and anthrax. The yearly fatality rate of each disease showed a downward trend.

Among the diseases with overall fatality rates of less than 1%, malaria had a yearly fatality rate after 2011 of between 0.50% and 0.70%, which was higher than the previous recorded rate of 0.02%-0.24%. The yearly fatality rates of some diseases were affected by a fewer number of cases or showed unexplained changes, as was the case for diphtheria, dengue, leprosy and typhus.

## DISCUSSION

Our analysis indicated that overall incidence and overall mortality of notifiable infectious diseases both showed a “U” distribution during 1986-2016, and the incidence of 22 persistently reported infectious diseases stabilized at a lower level after a rapid decline before 1993. Emerging and reemerging infectious diseases led to a rapid increase in the cases and incidence of notifiable infectious diseases from 2003 onwards.

From the 1960s to the 1980s, China experienced a large decline in the spread and burden of infectious diseases. With the increased implementation of control methods, infectious diseases declined year by year. In 1996, the incidence of notifiable infectious diseases dropped to its lowest value of 151.71/100 000. The reduction in the incidence at this time was a result of the continuous and large-scale public health interventions and large population-based vaccination programs that had been implemented. The rapid decline in the incidence of vaccine-controlled diseases was a result of planned vaccine immunization programs. For example, the incidences of diphtheria, pertussis, poliomyelitis and measles were significantly reduced as a result of the introduction in 1978 of a four vaccine program (BCG, polio trivalent vaccine, diphtheria and tetanus toxoid with acellular (DTP) vaccine and measles vaccine) targeting six diseases (tuberculosis, poliomyelitis, pertussis, diphtheria, neonatal tetanus, measles) [12]. During this period, vaccination against rabies, ECM and epidemic encephalitis B had not yet been included in the planned immunization, hence the decline in incidences of these three diseases could not only be attributed to vaccination. Living standards and the management of disease vectors and infection sources were improved and these public health measures also helped to reduce the incidence of natural epidemic diseases such as EHF, leptospirosis, malaria and typhus [3].

Since 2002, both the incidence and mortality of notifiable diseases showed a gradual upward trend. This trend is mainly due to the following reasons. First, as the notifiable disease report switched from paper-based reporting to internet-based reporting, the timeliness of reporting has improved. Second, an improvement in diagnostic levels for infections would affect incidence [13], which is attributable to great technological progress in laboratory detection and case identification. Moreover, the threat of infection continues to rise, due to increasing antimicrobial resistance, augmented population mobility, and changing human behavior.

After 2010, the incidence and mortality of notifiable diseases have slightly decreased. Various strategies [14] has been implemented to prevent the spread of infectious diseases after SARS epidemic in China. These strategies include improvements in the safety of blood collection, massive vector control, and early surveillance and warning with enhanced screening. Also, major special national science and technology projects on prevention and control were started at the end of 2008 [15].

Since the first reported HIV/AIDS cases [16] among domestic hemophiliac patients who received imported blood products, the HIV epidemic in China has expanded from high-risk groups to the general population and from rural regions to urban areas [17,18]. In the mid and late 1990s [19], infection among illegal blood donors had been eliminated, and HIV infection among injecting drug users had fallen, and remained stable at low levels among female sex workers. However, the yearly incidence of HIV/AIDS continued to rise after 2000 [17,20], and the increased openness of male homosexuality was reported to have played an important role in this increase [21]. There were 654 000 people living with HIV/AIDS in China by the end of September, 2016. The rise in the HIV/AIDS mortality rate may also be the result of years of accumulation of cases. Therefore, the yearly number of deaths and the yearly mortality rate may not be good markers for evaluating the burden of HIV/AIDS.

China ranks as having the second highest tuberculosis burden of any country in the world, accounted for 13% of the world’s cases of multidrug-resistant/RR-tuberculosis [22]. Progress in the control of tuberculosis was slow during the 1990s because of a malfunctioning health system [23]. After the SARS epidemic in 2003, the Chinese government implemented a series of measures to strengthen its public health system, which accelerated efforts to control tuberculosis. Tuberculosis is still among the top five diseases in terms of mortality rates and number of cases. Further strengthening of the public health system is needed, and a priority is to control the serious epidemic of multidrug-resistant tuberculosis in China.

Similar to HIV/AIDS and tuberculosis, the high mortality rate of viral hepatitis was related to the large number of cases, while the fatality rate was decreasing. Although the number of cases of hepatitis C was on the rise [24], the yearly incidence of viral hepatitis was at a stable high level [25], which was attributed to the fact that hepatitis B was still the predominant cause and its incidence was relatively stable. This mainly resulted from the implementation of HBV vaccine programs in China. The prevalence of hepatitis B among children has decreased dramatically, while its prevalence among adults remains high in China [26,27]. China also still faces the challenge of mother-to-child transmission of hepatitis B [28]. The number of reported deaths caused by hepatitis C also showed an upward trend [29]. In recent years, the number of hepatitis C virus infections among older adults has increased. Specific interventions and prevention programs targeting hepatitis C virus epidemic areas are therefore urgent. The incidence of hepatitis A decreased significantly year by year, and the risk has been reduced to a relatively low level.

The incidence of brucellosis is also rising rapidly [30], which is reported to be closely related to changes in cattle and sheep farming activities [31]. The occurrence of this disease is not affected by human cases. Medical institutions and disease control agencies can only detect and treat human cases, and then deduce the epidemic status in animals. Humans became “indicative cases” of an animal epidemic highlighting the problems in animal brucellosis prevention and control. Human brucellosis disease epidemics cannot therefore be prevented unless animal epidemics are under control. A similar situation exists with anthrax.

The high incidence and mortality rate of rabies suggests that there are also major problems in the prevention and control of this disease. During the whole study period, rabies was always among the top three causes of death from infectious disease, and the incidence has not decreased significantly. Human rabies cases were primarily a consequence of injures received from dogs in China [32,33], as seen in other regions of the world [34]. With the lack of effective dog management and the low vaccination rate of dogs, the unsuccessful control of canine rabies has been the main factor leading to the human rabies epidemic.

After a 20-year effort, sexually transmitted diseases were nearly eliminated in the 1970s [35]. However, since the start of socioeconomic reforms in 1978, immigration and commercial sex have increased dramatically and sexually transmitted diseases have been proliferating [35-37]. Syphilis incidence has increased [38], especially among homosexual men in China [39]. The incidence of gonorrhea also increased after 2014. Antimicrobial resistance among these pathogens is another area of concern for the future.

For infectious diseases with a high incidence, such as hand, foot and mouth disease, dysentery, influenza and other infectious diarrhea, the reporting of these diseases is affected by many factors, such as patient willingness, diagnostic criteria and the mode of management. The case numbers of these diseases were high, and the epidemic seasons were concentrated. Although internet-based reporting has been adopted, this reporting method still requires a lot of manpower and material resources. However, the reported data for these diseases has little to do with disease prevention and control. Therefore, national cytopathogenic surveillance systems have been established for influenza, and national pathogen surveillance systems have been established for dysentery and infectious diarrhea. Hence, it is necessary to further assess and change the modes of reporting for diseases as required.

Malaria, once a serious public health problem in China [40], has been initially controlled and a plan for elimination has been put in place. However, the number of deaths caused by imported falciparum malaria has increased in recent years.

Our analysis also found that some problems existed in national standardized reporting systems in China. Cases of schistosomiasis increased rapidly with an AAPC of 33.9% (95% CI = 10.0 to 62.9), which was related to a surge in the number of cases reported in 2015. In 2015, some provinces reported cases of positive blood tests in screening work, which were reported into the system. This suggests that the incidence rate (or reporting rate) of the notifiable infectious disease reporting system is greatly affected by diagnostic criteria. Similarly, the increase in the rate of covert syphilis is a result of improvements in diagnostic techniques and management practices. A defect in the reporting system was detected for rabies, which has a fatality rate of 91.06%, and the outcome of some cases may therefore not have been recorded. In China, there are still no reports of a cure for rabies.

This study comprehensively analyzed the notifiable infectious disease data for the 31 years from 1986 to 2016. The time span for this study was large, and the data could not therefore be obtained from other official way. During the study period, China has undergone tremendous changes [41], and the epidemic pattern of infectious diseases has also undergone considerable changes [42]. We believe that this analysis will benefit the future monitoring of infectious diseases and public health measures.

Reported yearly incidence could be underestimated as it is affected by screening intensity. Moreover, incidence could also be underestimated because of under-diagnosis and under-reporting, in which individuals with mild symptoms may not go to medical institutions and cannot enter the system. This study did not include new infectious diseases other than the notifiable reported infectious diseases, such as yellow fever [43], Zika virus disease [44] and fever with thrombocytopenia syndrome. Although the number of cases of these diseases is small, there are local outbreaks or imported cases. In addition, tsutsugamushi was removed from the list of notifiable infectious diseases in 1989, while there has been an upward trend in recent years, and outbreaks have even occurred in parts of southern China.
